# Novel 31-kHz calls emitted by female Lewis rats during social isolation and social inequality conditions

**DOI:** 10.1016/j.isci.2023.106243

**Published:** 2023-02-20

**Authors:** Shota Okabe, Yuki Takayanagi, Masahide Yoshida, Tatsushi Onaka

**Affiliations:** 1 Division of Brain and Neurophysiology, Department of Physiology, Jichi Medical University, 3311-1 Yakushiji, Shimotsuke-shi, Tochigi-ken 329-0498, Japan

**Keywords:** Biological sciences, Neuroscience, Behavioral neuroscience, Cognitive neuroscience

## Abstract

Whether commonly used experimental animals show aversion toward inequality of social rewards, as humans do remains unknown. We examined whether rats emitted the 22-kHz distress calls under social reward inequality. Rats showed affiliative behavior for a specific human who repeatedly stroked and tickled them. When experimenter stroked another rat in front of them and during social isolation, these rats emitted novel calls with acoustic characteristics different from those of calls emitted under physical stress, namely air-puff. Under inequality conditions, rats emitted calls with higher frequency (∼31 kHz) and shorter duration (<0.5 s) than those emitted when receiving air-puff. However, with an affiliative human in front of them, the number of novel calls was lower and rats emitted 50-kHz calls, indicative of the appetitive state. These results indicate that rats distinguish between conditions of social reward inequality and the presence of an experimenter, and emit novel 31-kHz calls.

## Highlights

• Ultrasonic vocalizations in rats exposed to social reward inequality were examined
• Rats showed affiliative behavior to a specific human who repeatedly touched them
• Rats emitted novel 31-kHz calls during isolation and social inequality conditions
• Rats could discriminate social inequality conditions

## Graphical abstract

**Figure:**
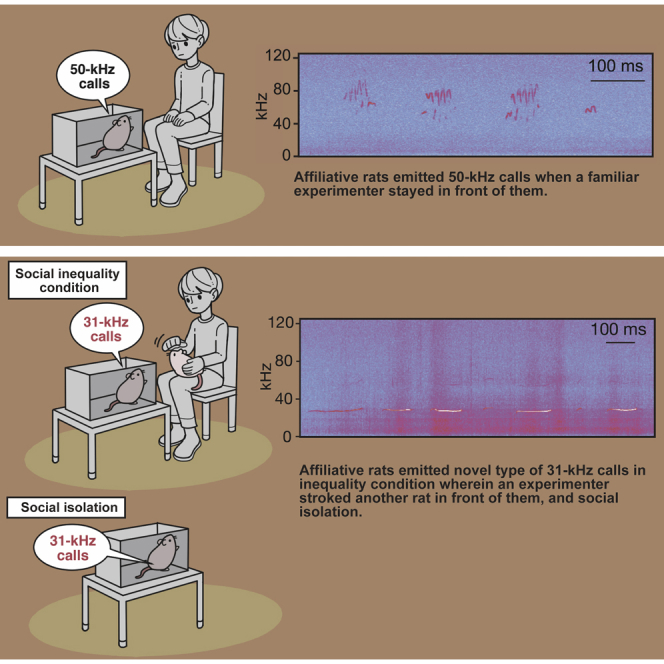

## Introduction

Humans show negative responses under situations of reward inequality. A sense of fairness and expression of disgust-related negative emotions toward reward inequality are considered to facilitate collaboration among cooperative group members.^1^^,^^2^ Recent research suggests that not only primates but also dogs^3^^,^^4^ and crows^5^ exhibit negative responses under inequality conditions. Rodents also exhibit negative responses under such conditions.^6^^,^^7^ These studies suggest that a sense of fairness is not specific to primates. In the majority of experiments on animals for the investigation of reward inequality, food has been used as a source of reward. Humans, however, show negative responses not only toward inequality of money and food reward but also toward inequality of social relationship-related reward. For example, they experience negative emotions—envy or jealousy—in social situations. However, little is known about neural mechanisms underlying responses to social reward inequality. Whether animals show negative emotion toward an inequitable distribution of social rewards remains unknown.

Previously, we found that rats receiving repeatedly stroked stimuli during their adolescence showed affiliative responses toward humans. They also preferred interacting with humans and stroking stimuli had a reward value that induced conditioned place preference.^8^^,^^9^ Humans feel jealous when affiliative specific persons show an affiliative attitude toward other persons. If rats can form an affiliative relationship toward a specific human, it may be possible to examine whether rats show a jealousy-like response; however, it is unknown whether rats show affiliative responses selectively toward specific humans.

Broadly, rats emit two types of ultrasonic vocalizations (USVs). USVs in the frequency range of 18–32 kHz, referred to as “22-kHz calls,”^10^^,^^11^ emitted under aversive states, such as exposure to predators, and those with a frequency greater than 35 kHz, referred to as “50-kHz calls,”^11^ emitted under appetitive or rewarding situations, such as social play. By analyzing USVs emitted under conditions of inequality, we can estimate whether rats demonstrate negative or appetitive state.

We investigated whether rats show negative states under conditions of social inequality. We first examined whether rats form an affiliative relationship with a specific human. Then, we recorded USVs to decipher whether rats emitted 22-kHz calls in a situation when an affiliative human stroked another rat. Oxytocin plays an important role in social relationships. Previously, we established a correlation between the activity of oxytocin neurons and affiliative behavior toward a human.^8^ We tested whether the inhibition of oxytocin function would disrupt affiliative relationships between rats and humans and prevent rats from exhibiting negative responses under social inequality conditions. While analyzing USVs emitted by rats under social inequality conditions, we noticed that these calls had acoustic characteristics (frequency, 26–40 kHz; duration, <0.5 s) different from those of the previously reported 50-kHz and 22-kHz calls. Depending on their frequencies and duration, we categorized the calls into three— categories depending on their frequencies and duration, 22-kHz calls (>18, ≤26-kHz, >0.5 s), 50-kHz calls (>40, ≤100-kHz, <0.5 s), and novel calls, 31-kHz calls (>26, ≤40-kHz, <0.5 s)—and analyzed the number of these calls during behavioral tests.

## Results

### Postweaning repeated affiliative touches facilitate affiliative behavior specific to a familiar experimenter in rats

**Figure 1:**
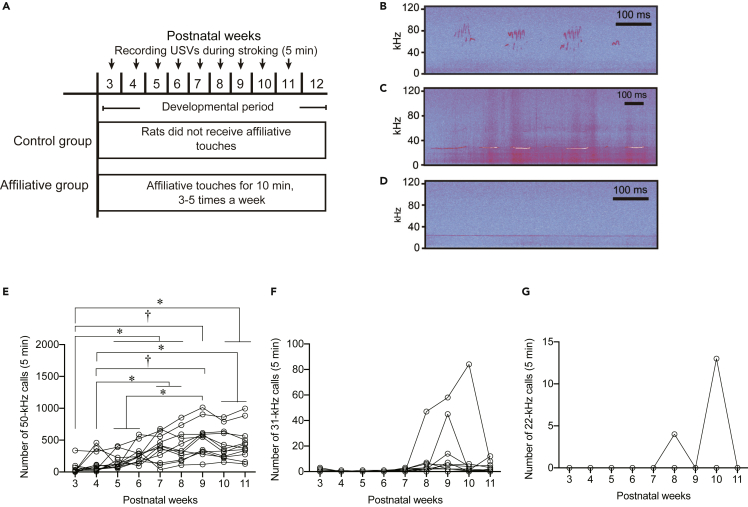
Postweaning repeated affiliative touches induced affiliative responsiveness in rats Time schedules of experiments. (A) Ultrasonic vocalizations were recorded for 5 min once per week between 3 and 11 weeks of age (developmental period) in the affiliative touch group. (B–G) (B) Spectrograms of 50-kHz, (C) 31-kHz calls, and (D) 22-kHz calls. Time courses of the numbers of vocalizations during stroking ((E), 50-kHz; (F), 31-kHz; (G), 22-kHz). †, p < 0.01, ∗, p < 0.05. Each line represents data for one individual rat.

To investigate whether female rats emit distress calls under an inequality social situation, we first produced affiliative female rats by giving gentle touches of stroking stimuli or tickling during the postweaning period according to procedures reported previously,^8^ with minor modifications. In the developmental period, we recorded USVs during stroking stimuli every week (Figure 1A) and counted the number of 50-kHz, 31-kHz, and 22-kHz calls (Figures 1B–1D) to confirm affiliative responsiveness (*N* = 12). Repeated measures one-way ANOVA revealed a significant effect of postnatal weeks of age [ *F* (8, 88) = 15.451, p < 0.001] in the number of 50-kHz calls. The number of 50-kHz calls at ages 5, 6, 7, 8, 9, 10, and 11 weeks was significantly increased compared with that at 3 weeks of age (3 weeks versus 5, 6, 7, 8, 9, 10, and 11 weeks, p = 0.026, 0.015, 0.013, 0.019, 0.006, 0.014, and 0.02, respectively) (Figure 1E). The numbers of 50-kHz calls at ages 7, 8, 9, 10, and 11 weeks were significantly increased compared with that at 4 weeks of age (4 weeks versus 7, 8, 9, 10, and 11 weeks, p = 0.023, 0.049, 0.009, 0.041, and 0.030, respectively). The number of 50-kHz calls at 9 weeks of age was significantly increased compared with that at 5 and 6 weeks of age (5 weeks versus 9 weeks, p = 0.022; 6 weeks versus 9 weeks, p = 0.031). The number of 31-kHz and 22-kHz calls during stroking was low and did not significantly change during development (Figures 1F and 1G). These data are consistent with previous findings that rats receiving stroking stimuli during the postweaning period show an affiliative response toward stroking stimuli.

**Figure 2:**
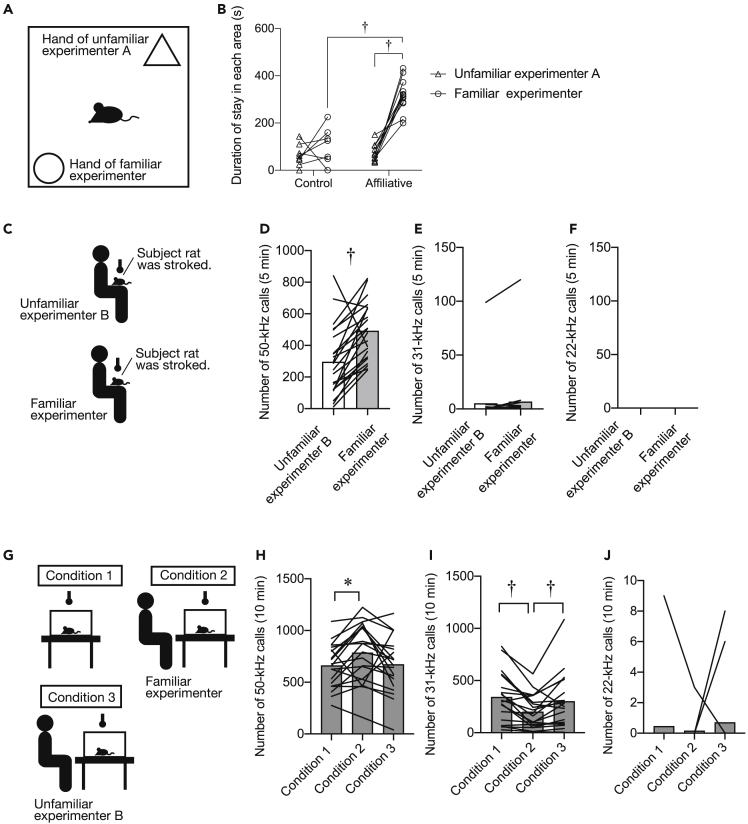
Postweaning repeated affiliative touches induce affiliative responsiveness toward a specific experimenter (A) Illustration of a social preference test. (B) Duration spent staying in the familiar experimenter’s hand area compared with that spent staying in the unfamiliar experimenter’s hand area in a social preference test. (C) Illustrations of experimental procedures. Ultrasonic vocalizations (USVs) were recorded when rats were stroked by an unfamiliar or familiar experimenter. (D–F) Number of 50-kHz (D) and 31-kHz (E), and 22-kHz (F) calls. (G) Illustrations of experimental procedures. USVs were recorded under the isolation condition (condition 1). USVs were also recorded when a familiar (condition 2) or an unfamiliar (condition 3) experimenter sat in front of the observer’s cage. (H–J) Number of 50-kHz calls (H) and 31-kHz calls (I) and 22-kHz calls (J). †, p < 0.01, ∗, p < 0.05. Each line or plot shows data for one individual rat.

Next, we examined whether rats show an affiliative response to a specific experimenter (Figure 2A, *N* = 12). Repeated measures two-way ANOVA revealed significant effects of group [*F* (1, 18) = 50.609, p < 0.001] and experimenter [*F* (1, 18) = 40.832, p < 0.001] and a significant interaction between the two [*F* (1, 18) = 24.827, p < 0.001] in time spent in hand areas in a preference test. The time spent staying in the familiar experimenter’s hand area was significantly longer than that in the unfamiliar experimenter’s hand area in the affiliative group (p < 0.001, post-hoc Holm’s test). The duration of stay in the familiar experimenter’s hand area in the affiliative group was also significantly longer than that in the control group (p < 0.001, post-hoc Holm’s test) (Figure 2B). Rats in the non-stroked control group did not show any preference (Figure 2B). These data indicate that rats in the affiliative group showed a preference toward a familiar experimenter.

We counted the number of USVs during stroking stimuli that were given by a familiar and an unfamiliar experimenter (Figure 2C, *N* = 22). The number of 50-kHz calls was significantly greater during stroking stimuli given by a familiar experimenter than that during stroking stimuli given by an unfamiliar experimenter (p = 0.001, Wilcoxon signed-rank test) (Figure 2D). The number of 31-kHz and 22-kHz calls did not differ significantly upon stroking stimuli by familiar and unfamiliar experimenters (Figures 2E and 2F).

Social isolation in an unfamiliar environment induces stress responses in social animals,^12^ and company of familiar individuals attenuates such responses.^12^^,^^13^ To determine whether rats exhibited negative states during isolation and if isolation-induced negative states were abolished by the presence of a familiar experimenter, we examined 22-kHz USV as an index of stress responses under isolation conditions and isolation with human presence (Figure 2G, *N* = 20). For isolation (condition 1), a subject rat was isolated in a clean cage. For isolation in the presence of a familiar experimenter (condition 2), a subject rat was kept in a clean cage and a familiar experimenter, who was sat in front of the cage, gave affiliative touches during the developmental period. In another condition of isolation in the presence of an unfamiliar experimenter (condition 3), a subject rat was kept in a clean cage and an unfamiliar experimenter sat in front of the cage. For the number of 50-kHz calls, repeated measures one-way ANOVA revealed a significant effect of condition [*F* (2, 38) = 4.451, p = 0.022]. The number of 50-kHz calls in the presence of a familiar experimenter (condition 2) was significantly greater than that in the social isolation condition (condition 1) (p = 0.028) (Figure 2H). Interestingly, for the number of 31-kHz calls, repeated measures one-way ANOVA revealed a significant effect of condition [*F* (2, 38) = 6.665, p = 0.009]. The number of 31-kHz calls in the presence of a familiar experimenter (condition 2) was significantly smaller than that in the social isolation (condition 1) or in the presence of an unfamiliar experimenter (condition 3) (condition 1 versus condition 2, p < 0.001; condition 3 versus condition 2, p = 0.019, post-hoc Holm’s test) (Figure 2I). The numbers of 22-kHz calls were not significantly different among the conditions (Figure 2J). These results showed that rats could discriminate between familiar and unfamiliar experimenters and are consistent with a view that repeated affiliative touches during adolescence resulted in the development of an affiliative relationship between rats and a familiar experimenter.

### Affiliative rats emit 31-kHz calls under a social inequality condition

**Figure 3:**
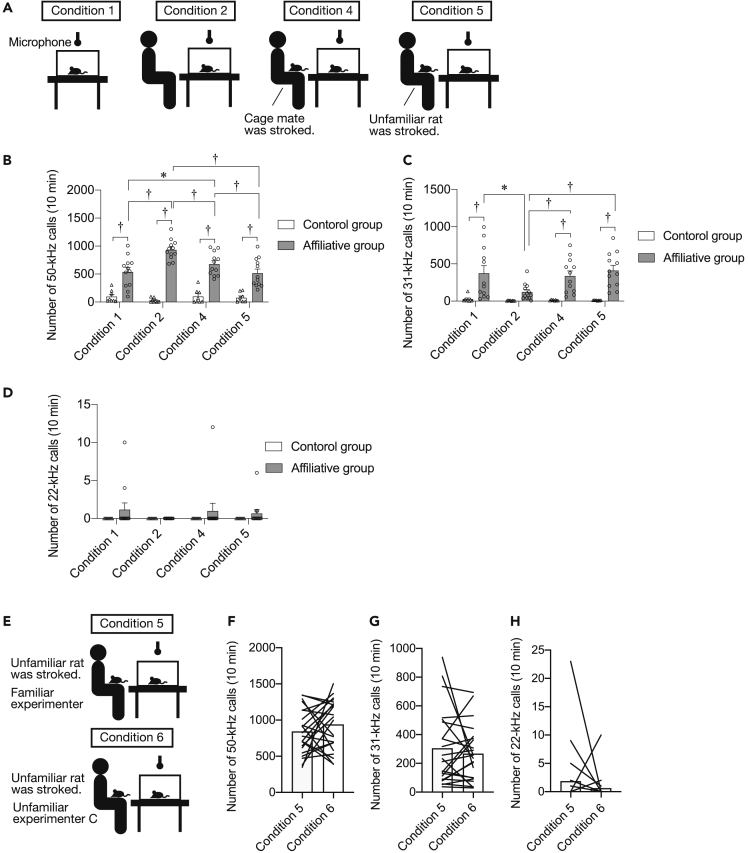
Affiliative rats emit 31-kHz calls under the inequality condition (A) Illustrations of experimental procedures. Ultrasonic vocalizations (USVs) were recorded in four conditions. In condition 1, subject rats were isolated in a clean cage. In condition 2, subject rats were kept in a clean cage and a familiar experimenter who had stroked the rats during the developmental period sat down in front of that cage. In condition 4, subject rats (observers) were kept in a clean cage and a familiar experimenter stroked cage mates (demonstrators) of subject rats in front of that cage. In condition 5, rats were kept in a clean cage and a familiar experimenter stroked unfamiliar rats (demonstrators) in front of that cage. (B–D) Number of 50-kHz (B), 31-kHz (C), and 22-kHz (D) calls under conditions 1, 2, 4, and 5. (E) Illustrations of experimental procedures. The observer rat’s USVs were recorded when a familiar (condition 5) or an unfamiliar (condition 6) experimenter stroked the demonstrator rat in front of the observer’s cage. (F–H) Number of 50-kHz (F) and 31-kHz (G), and 22-kHz (H) calls. †, p < 0.01, ∗, p < 0.05. Error bars denote SEM. Each line or plots shows data for one individual rat.

To determine whether rats emit 22-kHz calls under a social inequality situation, we examined the USVs under inequality conditions (affiliative group, *N* = 12; control group, *N* = 8). Rats were exposed to a social isolation condition (condition 1), isolation in the presence of a familiar experimenter (condition 2), or under inequality conditions wherein a subject rat (observer) was kept in a clean cage and a familiar experimenter sitting in front of the cage stroked its cage mate (condition 4) or an unfamiliar rat (condition 5) (Figure 3A).

Repeated measures two-way ANOVA revealed significant effects of group [*F* (1, 18) = 70.471, p < 0.001] and condition [*F* (3, 54) = 8.259, p = 0.001] on the number of 50-kHz calls and a significant interaction between the two [*F* (3. 54) = 14.123, p < 0.001]. The number of 50-kHz calls in the affiliative groups was significantly greater than that in non-stroked control groups in all conditions (in conditions 1, 2, 4, and 5, p < 0.001, post-hoc Holm’s test) (Figure 3B). The number of 50-kHz calls in condition 2 was significantly greater than that in the other three conditions (condition 2 versus conditions 1, 4, and 5, p < 0.001, = 0.001, and <0.001; post-hoc Holm’s test). The number of 50-kHz calls in the affiliative group in condition 4 was significantly greater than that in conditions 1 and 5 (condition 4 versus condition 1, p = 0.024; condition 4 versus condition 5, p = 0.002, post-hoc Holm’s test). The number of 50-kHz calls in the presence of the familiar experimenter was significantly reduced when the experimenter stroked another rat, especially an unfamiliar rat, suggesting that the presence of novel demonstrator rats reduced appetitive states.

Repeated measures two-way ANOVA revealed significant effects of group [*F* (1, 18) = 16.714, p = 0.001] and condition [*F* (3, 54) = 5.863, p = 0.007] on the number of 31-kHz calls and a significant interaction between the two [*F* (3. 54) = 5.068, p = 0.013]. The number of 31-kHz calls in the affiliative group was significantly greater than that of 31-kHz calls in the non-stroked control group in condition 1 (p < 0.001) and was significantly smaller in condition 2 than in condition 1 (condition 2 versus condition 1, p = 0.01, post-hoc Holm’s test). The isolation-induced increase in the number of 31-kHz calls was abolished by the presence of a familiar experimenter (Figure 3C). Interestingly, the number of 31-kHz calls in affiliative groups was increased in conditions 4 and 5 compared with the number of such calls in non-stroked control groups (Figure 3C, in condition 4 and 5, p < 0.001, post-hoc Holm’s test; Videos S1 and S2). The number of 31-kHz calls in affiliative groups in conditions 4 and 5 was also significantly greater than that in condition 2 (Figure 3C, condition 2 versus condition 4 and 5, p < 0.001, post-hoc Holm’s test). These results suggest that rats in the affiliative group show specific response to an inequality condition. No significant difference between the number of 31-kHz calls in conditions 4 and 5 suggests that the familiarity between observer and demonstrator rats is not crucial in the responsiveness to an inequality condition.

Video S1. 31-kHz calls emitted by observer rats when demonstrator rats received stroking stimuli in front of them, related to Figure 3 The experimenter stroked a demonstrator rat in front of the cage containing an observer rat. The audio signal provides ultrasonic vocalizations that have been transposed to the human audible frequency range.

Video S2. Rats in the affiliative group, but not those in the control group, emitted 31-kHz calls under an inequality condition, related to Figure 3 Distress calls of rats in the affiliative and control groups when the experimenter stroked a demonstrator rat in front of the cage containing an observer rat. The audio signal provides ultrasonic vocalizations that have been transposed to the human audible frequency range.

No significant difference among the conditions was noted in the number of 22-kHz calls (Figure 3D).

Envy and jealousy have long been regarded as distinct emotions.^14^ Envy occurs when a person recognizes the superior quality and achievement of other individuals. Unlike envy, jealousy occurs in the context of social relationships among three individuals. For example, a feeling of jealousy occurs when an affiliative familiar person directs his/her attention toward other persons but not to oneself. Thus, we also examined the effects of the familiarity of experimenters on the emission of 22-kHz calls by affiliative rats in another series of experiments. A subject rat (observer) was kept in a clean cage and a familiar (condition 5) or an unfamiliar (condition 6) experimenter stroked a demonstrator rat in front of it (Figure 3E, *N* = 22). There was no significant difference in the number of 50-kHz, 31-kHz, and 22-kHz calls of the observer rats between conditions 5 and 6 (Figures 3F–3H). The results suggest that familiarity with the experimenter did not significantly affect the emission of calls in the inequality condition. Further examination is needed to clarify the exact nature of emotion of rats emitting the calls.

### Acoustic characteristics of 31-kHz calls emitted under conditions of social isolation and inequality

**Figure 4:**
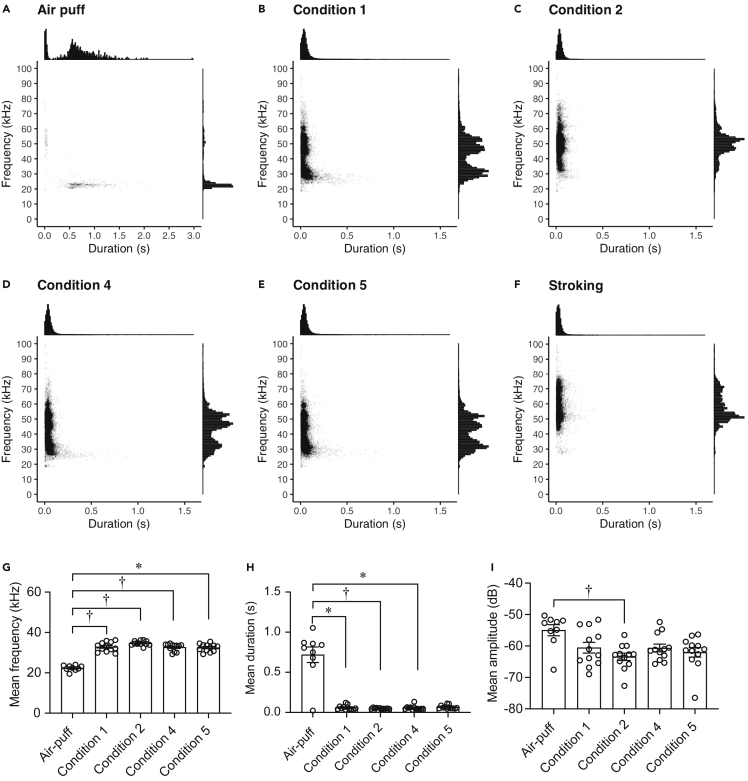
Acoustic characteristics of 31-kHz calls emitted under conditions of social isolation and inequality (A) Scatterplots and histogram of the number of ultrasonic vocalizations (USVs) for Lewis female rats (*N* = 9) under (A) air-puff stress. (B–I) USVs of affiliative group rats (*N* = 12) in (B) condition 1, (C) condition 2, (D) condition 4, (E) condition 5, and (F) stroking condition. (G) Mean frequencies, (H) durations, and (I) amplitudes of calls (frequency range below 40 kHz) under air-puff stress and conditions 1, 2, 4, and 5. †, p < 0.01, ∗, p < 0.05. Error bars denote SEM. Each scatterplot (A, B, C, D, E, F) shows individual calls from rats. Each plot in (G), (H), and (I) shows data for one individual rat.

Next, we investigated the acoustic characteristics of calls emitted during social situations compared with those under air-puff stress. Air puffs were applied to a separate series of non-stroked female rats (*N* = 9). Majority of USVs under air-puff stress had a low peak frequency of less than 26 kHz (Figure 4A). Contrarily, USVs emitted under social isolation (condition 1) or social inequality (conditions 4 and 5) had peak frequency of approximately 31 kHz and a mean frequency higher than that under the air-puff condition (Figures 4B, 4D, and 4E). In condition 2 and under stroking condition (familiar experimenter stroked affiliative rats), there were a large number of approximately 50 kHz calls (Figures 4C and 4F). The number of each call is shown in Table S1.

For analysis of the average frequency, duration, and amplitude, we combined all USVs below 40 kHz. The mean frequencies of calls (below 40 kHz) in social conditions 1 ((32.86 ± 0.67 (mean ± SEM, n = 12)), 2 (34.7 ± 0.33, n = 12), 4 (32.67 ± 0.52, n = 12), and 5 (32.45 ± 0.55, n = 12) were higher than that in the air-puff condition (22.38 ± 0.46, n = 9) (*χ*2 = 30.47, *df* = 4, p < 0.001, Kruskal-Wallis test, air-puff condition versus condition 1, 2, 4, and 5, p = 0.003, <0.001, = 0.006, and = 0.012, respectively, post-hoc Holm’s test) (Figure 4G). The mean duration of calls in conditions 1 (0.06 ± 0.008 (mean ± SEM, n = 12), 2 (0.047 ± 0.003, n = 12), 4 (0.057 ± 0.008, n = 12), and 5 (0.066 ± 0.007, n = 12) were shorter than that in the air-puff condition (0.722 ± 0.098, n = 9) (*χ*2 = 17.71, *df* = 4, p = 0.001, Kruskal-Wallis test, air-puff condition versus condition 1, 2, and 4, p = 0.018, 0.001, and 0.011, respectively, post-hoc Holm’s test) (Figure 4H). The mean amplitude of calls in social condition 2 (63.44 ± 1.172 (mean ± SEM, n = 12)) was lower than the amplitude in the air-puff condition (−54.97 ± 1.76, n = 9) (*χ*2 = 12.132, *df* = 4, p = 0.016, Kruskal-Wallis test, air-puff condition versus condition 2, p = 0.006) (Figure 4I). These results show that 31-kHz calls were acoustically different from the 22-kHz calls.

### An oxytocin receptor antagonist did not affect the social preference and emission of ultrasonic vocalizations under social inequality conditions

**Figure 5:**
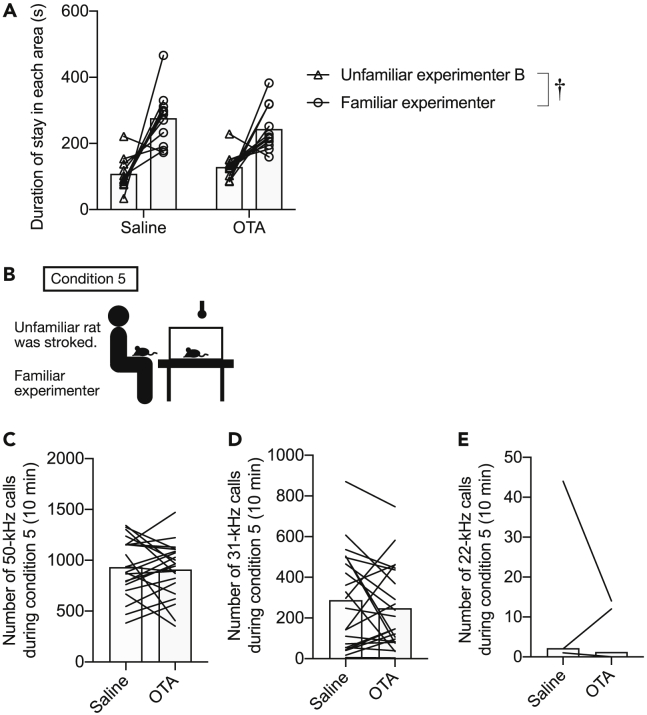
Oxytocin antagonist does not affect the social preference and emission of ultrasonic vocalizations (USVs) under inequality conditions (A) Duration of stay in the familiar and unfamiliar experimenter’s hand area in the social preference test after the administration of an oxytocin receptor antagonist (OTA) or saline. (B) Illustration of experimental procedures. USVs of observer rat were recorded 30 min after the administration of OTA or saline, when a familiar experimenter stroked unfamiliar rats in front of the observer’s cage. (C–E) Number of 50-kHz (C), 31-kHz (D), and 22-kHz (E) calls. †, p < 0.01. Each line or plot shows data for one individual rat.

We previously suggested that the affiliative response toward human or gentle stroking stimuli is correlated with the neural activities of oxytocin neurons in the caudal PVN.^8^ Thus, we examined the role of the oxytocin receptor in social preference toward an experimenter and emission of USVs under inequality conditions. A social preference test was conducted 30 min after intraperitoneal injection of an oxytocin receptor antagonist (OTA, *N* = 11) or saline (*N* = 11). Repeated measures two-way ANOVA revealed a significant effect of experimenter on the time spent in hand areas [*F* (1, 10) = 19.431, p = 0.001]. However, there was no significant effect of drug and interaction of the experimenter and drug (Figure 5A). Rats in the OTA-injected and saline-injected groups showed significantly higher preference toward a familiar experimenter than toward an unfamiliar experimenter. These results indicate that the oxytocin receptor does not play a crucial role in the expression of social preference toward a familiar experimenter. Moreover, there was no significant difference in the number of 50-kHz, 31-kHz, and 22-kHz calls under the social inequality condition in which other rats received stroking stimuli from a familiar experimenter between the antagonist-injected rats and saline-injected rats (Wilcoxon signed-rank test) (Figures 5B–5E, *N* = 22). This result suggests that the oxytocin receptor is not necessary for the emission of USVs under an inequality condition.

## Discussion

We demonstrate that rats that repeatedly received affiliative touches during the developmental period showed a specific social preference toward a familiar experimenter who repeatedly interacted with them. The number of 50-kHz calls when a familiar experimenter gently stroked the rats was greater than under the conditions when an unfamiliar experimenter stroked them. These results suggest that rats can distinguish between humans and exhibit increased appetitive states during stroking stimuli. A social bond has been defined based on behavioral and physiological responses—preference for the attachment figure, maintenance of proximity or voluntary contact with it, increased stress responses when separated from it, and decreased stress responses when reunited.^15^ We previously showed that repeated stroking stimuli induce behavior of approach and following toward a familiar human. Considering the behavioral responsiveness of affiliative rats in our previous and present study, it is possible that repeated stroking during the developmental period can result in social bonding between humans and rats. To investigate this, more experiments are needed including those that can help clarify the contribution of familiarity or habituation to affiliative behavior.

We show that affiliative rats emitted novel type of calls—31-kHz calls—during social isolation and under inequality conditions. These calls have shorter duration and higher frequencies compared with those of 22-kHz calls emitted under air-puff stress. Several studies revealed that young rats (4 weeks of age) emit 22-kHz calls with a higher frequency and shorter duration than do adult rats (11 or 12 weeks of age).^28^^,^^29^ In this study, the age of rats in the air-puff condition was 14 weeks, and the ages of rats in conditions 1–4 were 13–15 weeks. Thus, the difference in acoustic characteristics of 31-kHz calls was not due to age.^10^ Because rats produce USVs with acoustic characteristics different from those produced under air-puff stress, they might exhibit distinct emotions of social isolation and inequality that are different from those of invasive physical stimuli. We have no direct evidence to demonstrate the biological meaning of 31-kHz calls. However, the number of 31-kHz calls increased during the social isolation condition that is considered to induce stress responses in social animals. In contrast, in the presence of a familiar experimenter, the number of 31-kHz calls decreased whereas the number of 50-kHz calls increased. These data suggest that 31-kHz calls might reflect negative states. Brudzynski reported that 22-kHz calls can be subdivided into long- and short-forms of calls^30^ and that a short duration of 22-kHz calls was observed during withdrawal from cocaine.^31^ Previous studies suggested that the association of a negative state with an internal discontent is expressed by short calls.^11^^,^^32^ Therefore, it is suggested that short duration (<0.5 s) and high frequency (26–40 kHz) calls are associated with negative states. Wistar and Sprague-Dawley (SD) male juvenile rats were reported to emit approximately 30 kHz, short (0.3 s) calls during exposure to playback 50-kHz.^32^ For understanding the physiological meanings of 31-kHz calls, further studies are necessary to clarify the effects of strains and to list situations when rats emit 31-kHz calls.

In the present study, rats that repeatedly received affiliative touch stimuli emitted high number of 31-kHz calls under social isolation and inequality conditions, whereas non-touched control rats did not. These data suggest that 31-kHz calls are linked to social experience and social settings.

Rats emitted a larger number of 31-kHz calls under isolation and inequality conditions, but not in the presence of a familiar experimenter, suggesting that rats distinguished social conditions and might show negative states in socially isolated and social inequality conditions. The aversive property of inequitable outcomes has been demonstrated in several non-human primates^16^^,^^17^^,^^18^ and other social species including domestic dogs,^3^^,^^4^ corvids,^5^ and rodents.^6^^,^^7^ It is possible that inequality aversion and sociality, particularly cooperation, may have coevolved.^19^ Rats are a highly social species and form a hierarchically structured, well-organized social group. Several studies have suggested that rats show coordinated cooperative actions,^20^ reciprocity,^21^ empathy and prosocial behavior.^22^^,^^23^ Kashtelyan et al. reported that rats emitted 22-kHz calls during the observation of conspecific reward, food delivery.^24^ However, several studies have failed to demonstrate inequality aversion in social species.^25^^,^^26^^,^^27^ It has been argued that costly refusals of unfair offers, as demonstrated in several primate studies, may merely reflect nonsocial motives, such as frustration effects and/or violated expectations. However, frustration, expectation violations, or other nonsocial motives are unlikely to completely explain the emission of 31-kHz calls in our study. Rats in the affiliative group emitted a larger number of 31-kHz calls both during social isolation and under an inequality condition but not in the presence of a familiar experimenter (condition 2). If experimental environments including the existence of the experimenter induced the anticipation of social reward, rats should have emitted 31 -kHz calls under condition 2. We thought that nonsocial motives, such as betrayal of expectation, are unlikely to fully explain the emission of 31-kHz calls.

Studies concerning inequity aversion in animals have focused on behavioral responses, and the neural mechanisms underlying inequality aversion remain unclarified. Several studies suggest that dopamine, serotonin, and oxytocin modulate the egalitarian and trusting behaviors in humans.^33^^,^^34^^,^^35^ Oxytocin modulates affiliative social behaviors in animals.^36^ It modulates the responses to inequity in dogs.^37^ From these studies, we expected that the administration of oxytocin receptor antagonist would affect the response to an inequality condition in rats. However, contrary to our expectation, the oxytocin receptor antagonist did not significantly affect the emission of USVs under an inequality condition as well as under social preference behavior. It is possible that oxytocin contributes mainly to the building process of social relationships, not to the expression of affiliation-related responses. Further research is needed to clarify the neural mechanisms of inequality aversion.

### Limitations of the study

In this study, we did not conduct any experiments to investigate the biological meaning of 31-kHz calls. It is interesting to clarify the characteristics of emotional states including valence (positive or negative) in rats emitting 31-kHz calls. The identification of the biological meaning of 31-kHz calls will help us understand the responsiveness of rats to social inequality.

We used a single dosage of an oxytocin receptor antagonist, which blocks the activity of the oxytocin receptor, and found no significant effects. The present results are apparently inconsistent with the previously proposed idea that oxytocin plays an important role in an affiliative relationship between heterospecies.^8^ However, oxytocin systems have heterogeneity.^38^ Further experiments with photogenetic or chemogenic manipulation of the activity of specific oxytocin systems are necessary to elucidate the exact function of oxytocin in establishing an affiliative relationship between humans and rats and in the emission of 31-kHz calls.

In conclusion, we demonstrate for the first time that affiliative rats showed positive or appetitive states and a preference toward a specific human, and these rats emit novel 31-kHz calls under social isolation and inequality conditions. This study provides an experimental animal model to investigate molecular and neural mechanisms underlying affiliative relationships and inequality aversion.

## STAR★Methods

### Key resources table

**Table:** 

REAGENT or RESOURCE	SOURCE	IDENTIFIER
**Experimental models: Organisms/strains**		

Rat (LEW/CrlCrlj)	The Jackson Laboratory Japan, Inc.	https://www.jax.or.jp/

**Software and algorithms**		

Avisoft RECORDER	Avisoft Bioacoustics	https://www.avisoft.com/
SpectoLivellus 2D	Katou Acoustics Consultant Office	N/A
Audacity	The Audacity Team	https://www.audacityteam.org/
MATLAB	MathWorks	https://www.mathworks.com/products/matlab.html
USVSEG	Our previous study	https://sites.google.com/view/vocalcommuj/resource?authuser=0
Time OFCR1	O’Hara & CO., LTD	https://ohara-time.co.jp/
HAD	(Shimizu, 2016)^42^	https://osf.io/32cyp/
Prism8	GraphPad Software	https://www.graphpad.com/

### Resource availability

#### Lead contact

Further information and requests for resources should be directed to Shota Okabe (shota.okabe@jichi.ac.jp).

#### Materials availability

This study did not generate new unique reagents.

### Experimental model and subject details

#### Animals

Ninety-seven Lewis female rats (LEW/CrlCrlj, Charles River Laboratories Japan, Inc., Kanagawa, Japan) were used. Our previous studies^8^^,^^9^ reported that female Lewis rats emit more USVs than male rats. Thus, we investigated vocal responses of female rats in this study. The rats were obtained from an animal supplier at 3 weeks of age and were housed in pairs under a 12:12 h light/dark cycle (lights on at 7:30 am) at 22 ± 2°C and 55 ± 15% relative humidity. Food and water were available *ad libitum.* Animal experiments were conducted after receiving approval from the Animal Experiment Committee of Jichi Medical University and were in accordance with the Institutional Regulations for Animal Experiments and Related Activities in Academic Research Institutions under the Jurisdiction of the Ministry of Education, Culture, Sports, Science and Technology.

### Method details

#### General experimental design for preparing the animals

In the affiliative group, rats were kept in pairs and received affiliative touches 3 to 5 times a week from an experimenter (S.O.) for 4 weeks between 3 and 6 weeks of age. Affiliative touches consisted of affiliative interactions, such as stroking and tickling. Stroking stimuli were applied by an experimenter (S.O.), with the hand of the experimenter moving over the back of each animal at 5–10 cm/s. Tickling stimuli consisted of three phases—dorsal tickling, flip (rapidly overturned), and ventral tickling (vigorous tickling on the ventral trunk while the animal was pushed on the floor). Each phase lasted approximately 5–10 s and the duration of one tickling stimulus was 15–30 s. Tickling was repeated at 15 s intervals. A pair of rats received stroking stimuli simultaneously. The experimenter wore cotton gloves and a lab coat during interaction with rats. Rats in the control group (*N* = 8) were housed in pairs and did not receive affiliative touches until the time of the experiments. Another group of female rats, aged 14 weeks, was used for recording USVs under air-puff stress (*N* = 9). The rats in the control group were exposed to an experimenter during cage cleaning twice a week.

USVs in the affiliative group were recorded during a 5 min stroking session once each week during the developmental period (3–6 weeks of age). Rats were individually placed on the experimenter's (S.O.) lap and each rat was given massage-like stroking stimuli from the experimenter while on the experimenter’s lap. The number of 50-kHz calls (frequency between 40 and 100 kHz), 22-kHz calls (frequency between 18 and 26 kHz), and 31-kHz calls (frequency between 26–40 kHz) were automatically counted using our custom-written MATLAB codes.^39^ We segmented the calls based on a minimum silent duration of 40 ms. At 6 weeks of age, we selected rats for further experiments according to the total number of 50-kHz calls recorded at 3, 4, 5, and 6 weeks of age in response to stroking stimuli. We conducted two series of experiments and selected rats according to the number of 50-kHz calls. Previously, we showed that approximately 40% of rats emit a large number of USVs. We, therefore, selected the top 12 rats out of 28 in the first series and the top 22 rats out of 52 in the second series of experiments. These 34 rats received subsequent procedures and data for these were analyzed. The number of subjects was determined based on previous behavioral studies and considering the number of rats that could practically be treated in one series of experiments. Rats in the first series were used for experiments described in Figures 1A–1G, 2A, 2B, 3A–3D, and 4A–4I. Rats in the second series were used for experiments described in Figures 2C–2J, 3E–3H, and 5. These selected rats in the affiliative group received affiliative touches 3 to 5 times a week for another 6 weeks between 7 and 12 weeks of age. Thus, rats in the affiliative group received affiliative touches for 10 weeks in total between 3 and 12 weeks of age.

#### Unfamiliar experimenter

Three male experimenters took the role of unfamiliar experimenter in the following experiments: unfamiliar experimenter A in Figures 2A and 2B, unfamiliar experimenter B in Figures 2C–2J and 5A, and unfamiliar experimenter C in Figures 3E–3H.

#### General protocols for recording USVs

We used two types of microphones (Type 4158 N, Aco, Tokyo, Japan: CM16, Avisoft Bioacoustics, Berlin, Germany) and an A/D converter (Ultra-SoundGate, Avisoft Bioacoustics, SpectoLibellus 2D, Katou Acoustics Consultant Office, Kanagawa, Japan), which exhibit similar performance in recording USVs. None of the sets of microphones and converters caused any difference in USV analysis. The microphone was placed approximately 20 cm from the rats (see section "Recording of USVs during each condition).

#### Recording of USVs under each condition

Condition 1: Each rat was placed and kept in a clean cage that was the same as its home cage. USVs were recorded for 15 min.

Condition 2: Each rat was kept in a clean cage as in condition 1 and a familiar experimenter (S.O.) sat in front of the cage (approximately 60 cm from the cage). USVs were recorded for 15 min. The experimenter remained seated and tried not to move.

Condition 3: Each rat was kept in a clean cage as in condition 1 and an unfamiliar experimenter (B) sat in front of the cage (approximately 60 cm from the cage). USVs were recorded for 15 min. The experimenter remained seated and tried not to move.

Condition 4: Each rat was kept in a clean cage as in condition 1 and a familiar experimenter stroked its cage mate in front of the cage, approximately 60 cm from the cage. USVs were recorded for 15 min.

Condition 5: Each rat was kept in a clean cage as in condition 1 and a familiar experimenter (S.O.) stroked another rat (demonstrator) that was unfamiliar to the subject rat in front of the cage, approximately 60 cm from the cage. USVs were recorded for 15 min.

Condition 6: Each rat was kept in a clean cage as in condition 1 and an unfamiliar experimenter (C) stroked another rat (demonstrator) that was unfamiliar to the subject rat in front of the cage, approximately 60 cm from the cage. USVs were recorded for 15 min.

Air-puff condition: To elicit distress 22-kHz calls, another group of rats was individually transferred to an experimental cage and habituated for 5 min. Thereafter, each rat received an air-puff stimulus (0.3 MPa) with an inter-stimulus interval of 2 s at the nape from a distance of approximately 5–10 cm. USVs were recorded for 5 min immediately after delivering 30 air-puff stimuli. The data used in our previous study on the establishment of algorithms of software programs (USVSEG) for automatic analysis of USVs^38^ were used.

The number of 50-kHz calls decreased continuously during the first 10 min in affiliative rats under the isolation condition (condition 1) (Figure S1A. Changes in the number of ultrasonic vocalizations (USVs) over time, Related to Figure 3). In contrast to rats in the affiliative group, those in the control group emitted a smaller number of USVs and there were no significant changes in the number of calls (Figure S1B). Thus, we used calls for the first 10 min for analysis (condition 1 to 6).

#### Social preference test

A social preference test was used for assessing preference to the hand of the experimenter (S.O.) and that of an unfamiliar experimenter. The hands of the experimenter and an unfamiliar experimenter were placed at diagonally opposite corners of a test box (W 60 cm × D 60 cm × H 40 cm). A third experimenter put each animal in the test box at a corner different from the corners where hands were placed. The time spent in a square (20 × 20 cm) of the hand area was measured automatically during the 10 min test period using a program (Time OFCR1, O’Hara & CO., LTD, Tokyo, Japan). Both experimenters were blind to the treatments of subjects (group and identification). Different male experimenters performed the role of unfamiliar experimenter in each social preference test (unfamiliar experimenter A in Figure 2B and unfamiliar experimenter B in Figure 5A).

#### Intraperitoneal administration

A nonpeptidergic oxytocin receptor antagonist, L-368,899 (R&D Systems, MN, USA), was dissolved in 0.9% saline at a concentration of 0.2 mg/mL and administered to rats via intraperitoneal (IP) injection at a dose of 1 mg/kg body weight.^40^^,^^41^

#### Experimental schedule

USVs of selected rats in the affiliative group in the first series of sessions (*N* = 12) and rats in the control group (*N* = 8) were first recorded in condition 1 (13 weeks of age). On days 2 and 3, USVs of the rats were recorded in condition 2 (14 weeks of age). On days 4 and 5, USVs of the rats were recorded in condition 4 (14–15 weeks of age). Rats were housed in pairs and one of the pairs was assigned as the demonstrator and the other was assigned as the observer in condition 4. The experiments for condition 4 were conducted over a period of 2 days, and the order of the roles of observer and demonstrator was counterbalanced; the observer rats on day 4 were assigned as the demonstrators on day 5 and the demonstrator rats on day 4 were assigned as observer rats on day 5. On day 6, rats were examined for social preference toward experimenters in the social preference test (15 weeks of age). On the last day, USVs of rats were recorded in condition 5 (17 weeks of age). All the tests were performed for all the rats.

Selected rats in the affiliative group in the second series of sessions (*N* = 22) were first examined for social preference toward experimenters in the social preference test on day 1 (14 weeks of age). On days 2 and 3, USVs of rats were recorded in conditions 5 and 6 (14–15 weeks of age). The experiments were conducted over a period of 2 days, and the order of experimenters was counterbalanced. On days 4 and 5, USVs of half of the rats (*N* = 11) were recorded in condition 5 at 30 min after IP administration of an oxytocin receptor antagonist or saline (16 weeks of age). The experiments were conducted over 2 days, and the order of drugs was counterbalanced. On days 6 and 8, the same rats (*N* = 11) were tested for social preference, 30 min after IP administration of an oxytocin receptor antagonist or saline (16 weeks of age). The experiments were conducted over a period of 2 days, and the order of drugs was counterbalanced. On days 7 and 9, USVs of the remaining rats (*N* = 11) were recorded in condition 5 at 30 min after IP administration of the oxytocin receptor antagonist or saline (16 weeks of age). The experiments were conducted over a period of 2 days, and the order of drugs was counterbalanced. Thus, the total number of animals in condition 5 with drug administration was 22, and the total number of animals in the social preference test with drug administration was 11. On day 10, USVs of all rats were recorded in condition 1 (21 weeks of age). On days 11 and 12, USVs of rats were recorded in conditions 2 and 3 (21–22 weeks of age). The experiments were conducted over a period of 2 days, and the order of conditions was counterbalanced. The number of rats was 20 due to a problem with the equipment. On days 13 and 14, USVs of rats were recorded during stroking stimuli by a familiar or an unfamiliar experimenter (22–23 weeks of age). The unfamiliar experimenter learned the stroking procedures before experiments. IP administration was conducted by an experimenter blind to treatments of the animals and the experimenter of behavioral tests was blind to treatments of IP administration.

### Quantification and statistical analysis

Statistical analysis was performed using a free software, HAD.^42^ Developmental changes in the numbers of USVs in the affiliative group (Figures 1E–1G), time course of the number of USVs for 15 min in condition 1 (Figures S1A–S1D), and number of USVs during conditions 1, 2, and 3 (Figures 2H–2J) were analyzed by repeated-measures one-way ANOVA followed by post-hoc Holm's test. Results of the social preference test shown in Figures 2B and 5A were analyzed using repeated two-way ANOVA (group × experimenter, drugs × experimenter) followed by post-hoc Holm’s test. The number of USVs during conditions 1, 2, 4, and 5 (Figures 3B–3D) was also analyzed using repeated two-way ANOVA (group × condition). Acoustic characteristics in the air-puff condition and in conditions 1, 2, 4, 5, and stroking condition were analyzed by the Kruskal–Wallis test followed by post-hoc Holm’s test (Figures 4G–4I). Differences in the number of USVs between a familiar experimenter and an unfamiliar experimenter (Figures 2D–2F) and between conditions 5 and 6 (Figures 3F–3H) were analyzed using the Wilcoxon’s signed-rank test. The number of USVs after administering drugs was also analyzed using the Wilcoxon’s signed-rank test (Figures 5C–5E). *P* < 0.05 was considered statistically significant.

## Data Availability

The datasets supporting the current study have not been deposited in a public repository but are available from the corresponding author on request. This study did not generate code.
