# Supplementary material for: Novel 31-kHz calls emitted by female Lewis rats during social isolation and social inequality conditions
Source: iScience. 2023 Feb 20;26(3):106243. doi: 10.1016/j.isci.2023.106243 (PMC10009291; doi:10.1016/j.isci.2023.106243)
Supplement: Document S1. Figure S1 and Table S1 [file mmc1.pdf]

iScience, Volume *26*

Supplemental information

Novel 31-kHz calls emitted by female Lewis rats during social isolation and social inequality conditions

Shota Okabe, Yuki Takayanagi, Masahide Yoshida, and Tatsushi Onaka

Supplemental information

Figure S1. Changes in the number of ultrasonic vocalizations (USVs) over time, related to Figure 3.

Table S1. Acoustic characterisitics of USVs emitted under several conditions, related to Figure 4.

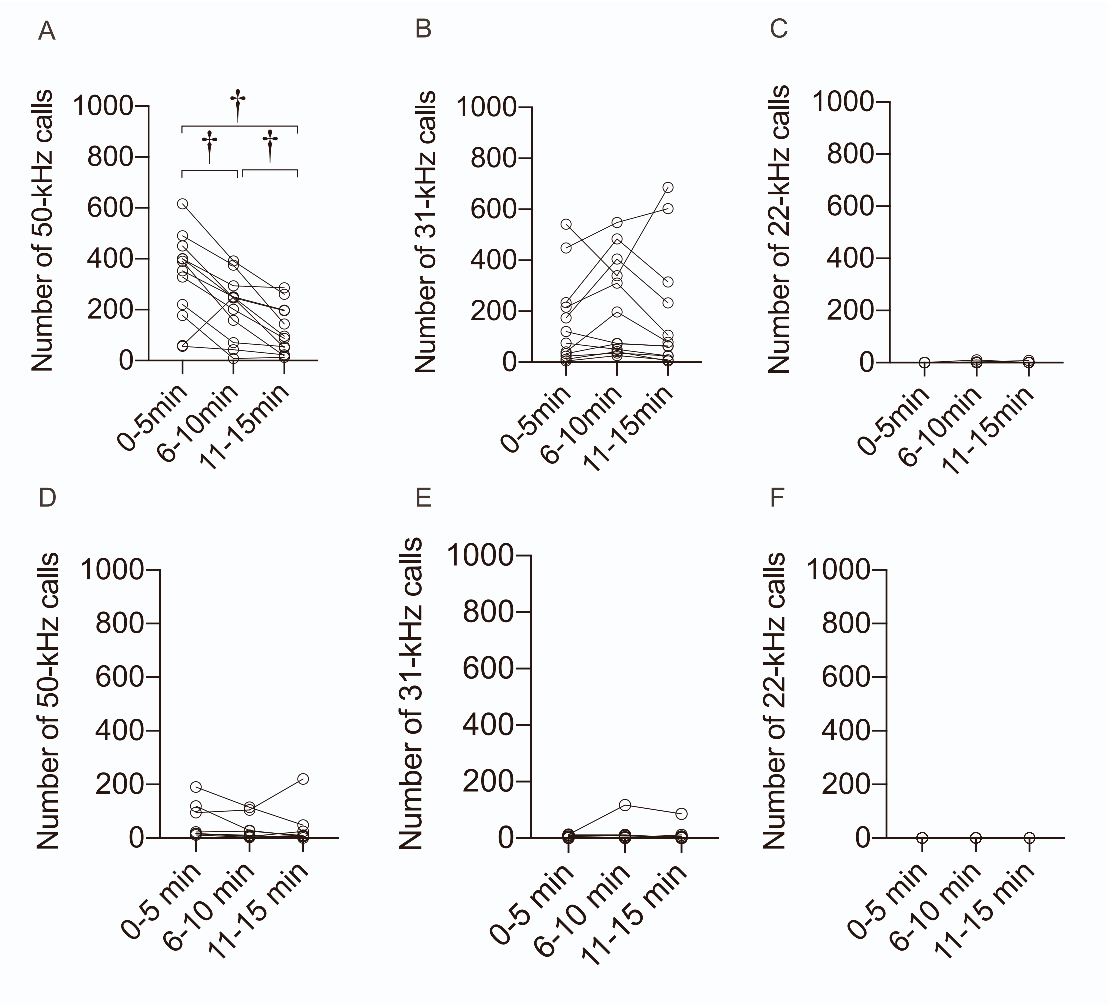

Figure S1. Changes in the number of ultrasonic vocalizations (USVs) over time, related to Figure 3.

Time courses of the numbers of vocalizations in the affiliative group (A, 50-kHz; B, 31-kHz; C 22-kHz) and control group (D, 50-kHz; E, 31-kHz; F 22-kHz) under condition 1. For the number of 50-kHz calls in the affiliative group, repeated measures one-way ANOVA revealed a significant effect of time [F (2, 22) = 20.726, $P < 0.001$]. The number of 50-kHz calls in 6–10 min and in 11–15 min was significantly smaller than the number of their number in 0–5 min (0–5 min versus 6–10 min, $P = 0.004$; 0–5 min versus 11–15 min, $P = 0.001$, post-hoc Holm's test). The number of 50-kHz calls in 11–15 min was significantly smaller than that in 6–10 min ($P = 0.004$, post-hoc Holm's test). †, $P < 0.01$.

			Frequency (kHz)			
Condition			>18, = or <26	>26, = or <40	>40, = or <100	Total calls
Air-puff	Durations	<0.5	53 (8.51%)	12 (1.93%)	93 (14.93%)	623
		>0.5	451 (72.39%)	11 (1.77%)	3 (0.48%)	
	USV frequency most numerously detected (number of syllables)		22 kHz (232)	28 kHz (5)	58 kHz (9)	
Condition1 (Isolation)	Durations	<0.5	322 (2.84%)	4510 (39.73%)	6476 (57.05%)	11351
		>0.5	14 (0.12%)	28 (0.25%)	1 (0.01%)	
	USV frequency most numerously detected (number of syllables)		26 kHz (232)	31 kHz(585)	46 kHz (499)	
Conditon2	Durations	<0.5	38 (0.30%)	1492 (11.71%)	11211 (87.98%)	12741
		>0.5	0 (0.00%)	0 (0.00%)	0 (0.00%)	
	USV frequency most numerously detected (number of syllables)		26 kHz (13)	35 kHz (174)	52 kHz (1041)	
Condition4 (Inequality)	Durations	<0.5	346 (2.75%)	4078 (32.35%)	8157 (64.72%)	12602
		>0.5	12 (0.10%)	9 (0.07%)	0 (0.00%)	
	USV frequency most numerously detected (number of syllables)		26 kHz (229)	32 kHz (478)	46 kHz (671)	
Condition5 (Inequality)	Durations	<0.5	264 (2.31%)	4957 (43.31%)	6201 (54.18%)	11445
		>0.5	8 (0.07%)	15 (0.13%)	0 (0.00%)	
	USV frequency most numerously detected (number of syllables)		26 kHz (181)	31 kHz (656)	52 kHz (518)	
Stroking	Durations	<0.5	2 (0.02%)	148 (1.35%)	10807 (98.62%)	10958
		>0.5	0 (0.00%)	0 (0.00%)	1 (0.01%)	
	USV frequency most numerously detected (number of syllables)		26 kHz (9)	40 kHz (46)	52 kHz (1089)	

Table S1. Acoustic characterisitics of USVs emitted under several conditions, related to Figure 4.
